# BUILDING AND SUPPORTING RESILIENT COMMUNITIES FOR OLDER ADULTS

**DOI:** 10.1093/geroni/igae098.1010

**Published:** 2024-12-31

**Authors:** Elana Kieffer

**Affiliations:** The New York Academy of Medicine, New York, New York, United States

## Abstract

2023 was Earth’s warmest year on record, and the planet’s temperatures will only continue to rise. Older adults are among those most vulnerable to the intense and increasingly dangerous effects of climate change. Extreme heat and extreme cold can cause significant health consequences, particularly for older adults, that impact their body temperatures, respiratory systems, levels of exhaustion, and even survival. In addition, weather disasters such as hurricanes and floods are on the rise due to climate change. Not only do they pose immediate threats to the safety and well-being of older adults, but they contribute to prolonged challenges in the aftermath, including disruptions to essential services, displacement of individuals and families, and compromised access to healthcare. In this session, the Center for Healthy Aging at The New York Academy of Medicine (NYAM) will describe how older New Yorkers and the professionals who work with them can build resilient response systems, strategies, and programs in the face of these challenges, as outlined in “Strengthening Community Resilience: Supporting Older Adults Through Emergency Preparedness And Response In A Post-COVID Era”, published by NYAM in 2023. We will discuss recommendations and strategies in four key areas: partnerships, education, efficiency of services, and access to services. Building and supporting resilient communities with a focus on the strengths and needs of older adults is imperative in the fight against climate change, and we will outline concrete ways to achieve this objective.

